# Proteomic trajectories in human rotator cuff degeneration: a systematic review of immunohistochemical studies

**DOI:** 10.1186/s13018-026-06735-1

**Published:** 2026-02-09

**Authors:** Dave Osinachukwu Duru, Andrew Kailin Zhou, Patrick J. Carroll, Amr Elmaraghy

**Affiliations:** 1 School of Clinical Medicine, University of Cambridge, Cambridge, UK; 2 University of Warwick, Coventry, UK; 3 Department of Trauma and Orthopaedics, University Hospital Coventry and Warwickshire, Coventry, UK; 4 Division of Orthopaedic Surgery, Department of Surgery, University of Toronto, Toronto, ON Canada; 5 Surgeon Investigator, Li Ka Shing Knowledge Institute, Unity Health Toronto, Toronto, ON Canada

**Keywords:** Rotator cuff tear, Immunohistochemistry, Proteins, Hypoxia, Inflammation, Fibrosis, Biomarkers, Regenerative medicine

## Abstract

**Background:**

Rotator cuff (RC) repairs heal unreliably, particularly in chronic disease. The molecular mechanisms underlying failed repair remain poorly understood. While individual studies have examined protein expression in diseased RC tissue, no synthesis has investigated these findings across various disease stages. This systematic review explores the stage-specific protein expression of human RC degeneration using immunohistochemistry.

**Methods:**

Following PRISMA guidelines, MEDLINE, Embase, and Cochrane Library were systematically searched to September 2025 for human studies that use immunohistochemistry to evaluate protein expression in intraoperative RC tendon or muscle biopsies. Study quality was appraised with Joanna Briggs Institute (JBI) tools. No meta-analysis was performed due to heterogeneity.

**Results:**

Forty-seven studies were included. Despite methodological heterogeneity, convergent molecular patterns emerged within disease stages. Partial and small tears demonstrated hypoxic-inflammatory-apoptotic signatures (HIF-1α, BNip3, IL-6, IL-1β, MMP-1/3/9) with preserved regenerative markers (Ki67, CD34), suggesting reparative potential. Medium tears exhibited sustained angiogenic activity (VEGF) and emerging adipogenic drift (PPARγ, C/EBPα). Large and massive tears showed depletion of anabolic factors (TGF-β1, BMP-5), M2-macrophage predominance (CD206, CD163), and fibrofatty infiltration. Patient comorbidities (diabetes, vitamin D deficiency, smoking) amplified inflammatory and adipogenic signatures.

**Conclusions:**

Cross-sectional human immunohistochemical evidence infers a stage-associated molecular trajectory: inflammatory-hypoxic stress with retained reparative capacity at earlier disease stages to fibrotic–adipogenic failure at later stages. This molecular framework may support future approaches to surgical decision-making and precision therapies.

**Supplementary Information:**

The online version contains supplementary material available at 10.1186/s13018-026-06735-1.

## Background

Rotator cuff tears are a significant cause of shoulder dysfunction, affecting approximately 20% of individuals over 60 years of age [1]. The progression of rotator cuff disease from intact tendinopathy to full-thickness tears involves degenerative changes, such as muscle atrophy, fibrosis and fatty infiltration [2, 3]. Rotator cuff tears can be treated non-operatively by injections or physiotherapy, or via surgical repair [4]. Open, mini-open, and arthroscopic rotator cuff repairs generally lead to good clinical outcomes [5–7]. However, at times, outcomes following cuff repair may be suboptimal, particularly for those of lower socioeconomic status or cases of chronic massive tears, characterised by high re-tear rates [8–10]. While macroscopic patterns are visually clear, there remains a need to better understand the molecular basis of rotator cuff disease progression and failed repair healing [1].

In recent years, researchers have turned to gene expression profiling to elucidate the pathophysiology of rotator cuff disease. Numerous studies have investigated transcriptomic changes across disease stages and healing outcomes [11–15]. While transcriptomic analyses quantify upstream gene regulation, protein expression more closely reflects the downstream functional tissue state. As such, proteins may provide a more faithful representation of the active disease phenotype and intrinsic healing potential. To investigate this, studies have harnessed immunohistochemistry, enabling spatially resolved assessment of protein expression within rotator cuff tissue [16]. These studies highlight pathological changes in expression of proteins involved in extracellular matrix remodelling, inflammation, metabolism, and cell death [17]. Such studies were synthesised in prior histological and immunohistochemical reviews of rotator cuff disease [18, 19]. However, existing syntheses largely describe findings in aggregate and do not investigate protein expression at increasing stages of disease progression. Such progression is relevant to the concept of a *proteomic trajectory*, originally defined as the kinetic evolution of protein expression over biological time [20]. Although first applied to developmental transitions, this framework is well-suited to modelling the molecular progression of rotator cuff disease. Given the cross-sectional nature of immunohistochemical studies, any proposed molecular “trajectory” represents an inferential, stage-aligned framework, rather than a directly observed temporal sequence.

This review synthesises immunohistochemical evidence from human intra-operative tendon and muscle biopsies to characterise protein expression at various stages of rotator cuff disease. Designed a priori, it aims to delineate stage-specific molecular signatures of rotator cuff disease and examine the influence of patient comorbidities on these proteomic profiles.

## Methods

This systematic review was conducted in accordance with the Preferred Reporting Items for Systematic Reviews and Meta-Analyses (PRISMA) guidelines. A completed PRISMA checklist is provided as Supplementary Material. The review protocol was defined a priori, including the objectives, inclusion criteria, methods for study selection, data extraction, and analysis.

### Search strategy

A comprehensive literature search of MEDLINE (PubMed), Embase, and Cochrane Library databases was performed from inception to September 2025 to identify studies evaluating protein expression in diseased rotator cuff tissue using immunohistochemistry. The search strategy combined controlled vocabulary (MeSH/Emtree) and free-text terms relating to the rotator cuff (“rotator cuff”, “supraspinatus”, “infraspinatus”, “teres minor”, “subscapularis”) and immunohistochemical protein expression (“protein expression”, “cytokine”, “transcription factor”, “immunohistochemistry”, “immunohistochemical”, “immunofluorescence”, “immunofluorescent”). Boolean operators (“AND”, “OR”) were applied as appropriate, and search syntax was tailored for each database. The strategy was developed with a medical librarian and is provided in the Supplementary Materials. No restrictions were placed on publication type or status, and only studies published in or translated into English were included.

Additionally, reference lists of included studies and recent reviews were searched to identify additional records.

## Study selection

The following inclusion criteria were defined for this review:

*Population* Adults with clinically, radiographically, or intra-operatively confirmed rotator cuff disease. Studies examining comorbidity effects were eligible.

*Specimens* Intra-operative biopsy samples of rotator cuff tendon or muscle.

*Studies* Eligible studies reported quantitative or qualitative protein expression measured by immunohistochemistry. Studies with additional transcriptomic data were included only if immunohistochemistry outcomes were extractable. Case-control studies comparing torn vs. intact healthy tissue, cross-sectional analyses of protein expression in patient samples, or cohort studies were included. Both prospective and retrospective analyses were eligible. A minimum sample size of at least two participants was required.

*Exclusion criteria* Non-human, cadaveric, or cell-culture studies; mixed cohorts where rotator cuff data were non-extractable; and studies focused exclusively on other shoulder structures (subacromial bursa, capsule, or biceps tendon) without corresponding rotator cuff tissue. Investigations limited to genomic, DNA polymorphism, or transcriptomic data without immunohistochemistry assessment were excluded. Review articles, case reports, conference abstract, and editorials were omitted but screened for additional references.

After running the search queries, all citations were imported into Rayyan software and duplicates were removed. Two reviewers (D.O.D. and A.K.Z.) independently screened the titles and abstracts of all retrieved references for relevance. Studies that did not meet the inclusion criteria were excluded at this stage. For studies appearing potentially relevant, or where eligibility was uncertain, the full-text article was obtained. The two reviewers (D.O.D. and A.K.Z.) then independently assessed each full-text paper against the inclusion/exclusion criteria. Any disagreements in study selection were resolved through discussion, or by consultation with a third reviewer (P.J.C. or A.E.) if needed. The study selection process is summarised in a PRISMA flow diagram.

## Data extraction and synthesis

A standardised data extraction form was developed a priori to capture all relevant information from each included study. The following data were extracted:

*Study details* First author, publication year, country, and study design.

*Patient population* Sample size, patient demographics (age, sex), and any pertinent clinical details (tear size, chronicity, presence of comorbidities, and the operation the patient was undergoing).

*Tissue source and pathology* Anatomical site (tendon or muscle), tissue origin (supraspinatus, infraspinatus, subscapularis, teres minor), and pathological classification (intact histologically diseased tendon, partial-thickness tear, full-thickness tear). Rotator cuff disease severity was operationalized using standardized thresholds: intact tendinopathy, partial-thickness tear, small full-thickness tear (< 1 cm), medium full-thickness tear (1–3 cm), large full-thickness tear (3–5 cm), and massive full-thickness tear (> 5 cm). These severity categories are author-defined constructs imposed retrospectively. Studies that did not report the operationalised characteristics, or had ambiguous classification, were not assigned to severity-specific categories and were excluded from the staged narrative synthesis. Their findings were retained in the summary table to ensure comprehensive presentation of the available evidence (Table 1). Where differences between disease stages (e.g. small vs. large tears) are discussed in the Results, these comparisons are restricted to within-study analyses. In these studies, various tear sizes were comparatively examined within the same patient cohort using identical immunohistochemical methodology. No cross-study or temporal comparisons were performed. The presence and nature of comparison or control samples were documented.

*Immunohistochemistry methodology* The antibody targets, visualization system, and staining protocols were extracted. This included the type of detection system (e.g., horseradish peroxidase–diaminobenzidine [HRP–DAB], Vectastain ABC, LSAB, EnVision FLEX, or OMNIS platforms), secondary antibody characteristics, antigen retrieval methods (e.g., citrate buffer pH 6.0, enzymatic digestion), and any use of dual-labelling immunofluorescence.

*Protein expression outcomes* The direction of expression change (increased, decreased, or unchanged) in diseased versus control tissue, and the anatomical localisation of staining (tenocytes, fibroblasts, vascular endothelium, inflammatory cells, or muscle fibres) were extracted, where reported. Particular attention was given to the biological roles and functional categories of each protein to enable thematic synthesis of the findings. Reported correlations between protein expression and clinical or morphological parameters (tear size, chronicity, fatty infiltration, or postoperative healing potential) were noted.

As part of the data extraction, risk of bias was assessed independently by two reviewers (D.O.D. and A.K.Z.) using the Joanna Briggs Institute (JBI) critical appraisal tools appropriate to the study design. Disagreements were resolved by consensus or a third reviewer (P.J.C. or A.E.).

Given the heterogeneity across the included studies, a quantitative or semi-quantitative meta-analysis was not undertaken. Specifically, studies varied in (i) rotator cuff tear classification systems, with disease severity defined inconsistently using tear size, tendon retraction, fatty infiltration, symptom duration, or combinations thereof; (ii) immunohistochemical methodologies, including antibody selection, antigen retrieval protocols, detection systems, scoring approaches, and thresholds for defining “up-” or “down-” regulation; and (iii) choice of control tissue, which ranged from intact supraspinatus to subscapularis tendon, deltoid muscle, or hamstring specimens, each with distinct baseline protein expression profiles. In addition, most studies did not report standardized effect measures or variance estimates (e.g. fold change, standardized mean differences, or confidence intervals), precluding transformation to a common quantitative metric. Reported outcomes were therefore not statistically comparable across studies. Collectively, these inconsistencies violated the core assumptions required for valid quantitative pooling and risked generating misleading summary estimates.

Instead, a narrative synthesis was performed. Extracted data were organised thematically by disease severity. Consistent within study protein upregulation or downregulation and shared signalling pathways were identified and synthesised to evaluate concordance or conflict between studies. In addition, evidence relating specific proteomic signatures to clinical outcomes were presented where available. The reported trends represent directional changes within studies, rather than quantitative differences across the literature.

## Results

### Study characteristics

**Fig. 1:**
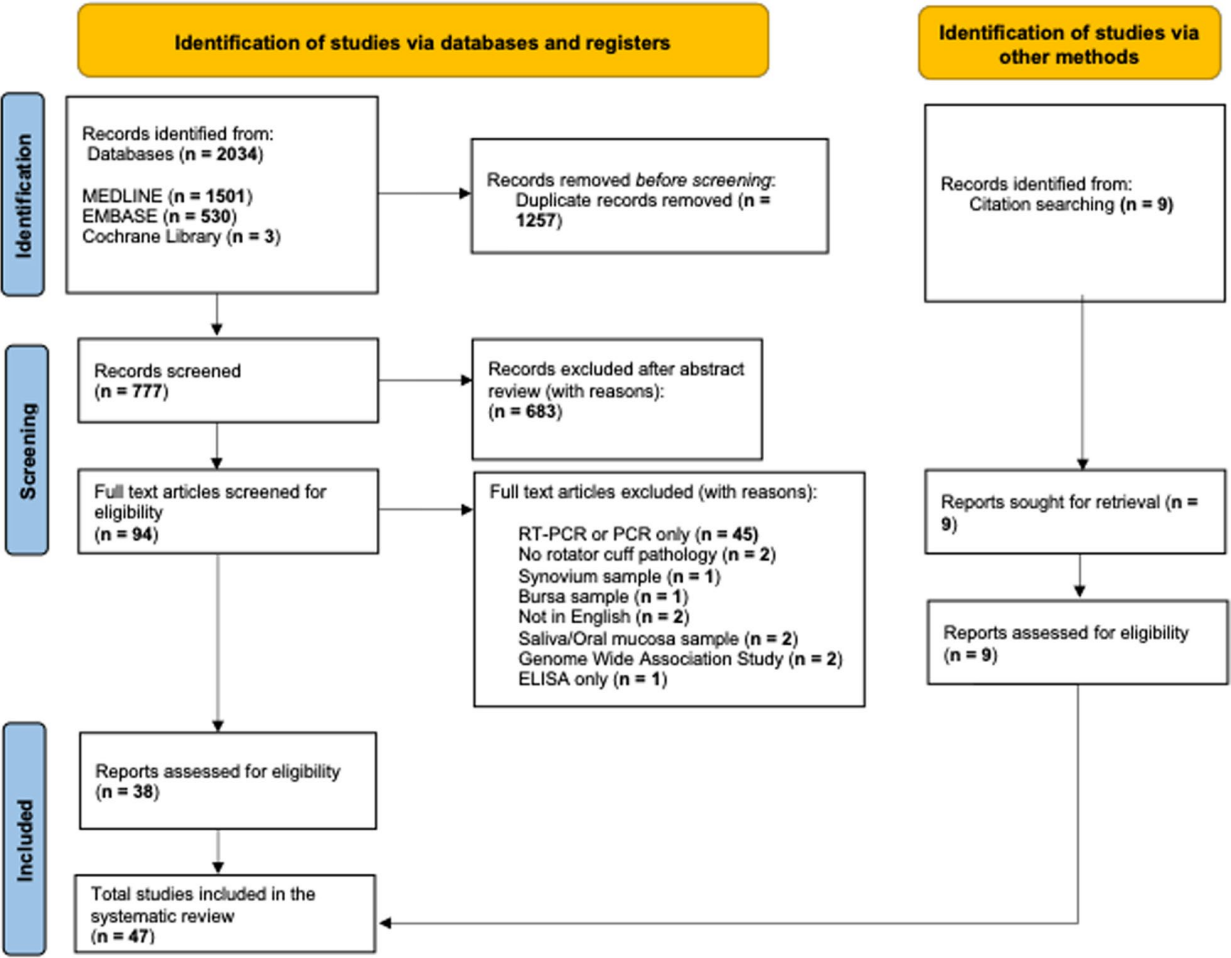
PRISMA Flowchart

Our search yielded 777 records after removing duplicates (Fig. 1). Forty-seven studies met the inclusion criteria (Table 1). Most samples were obtained intraoperatively as tendon biopsies during arthroscopic rotator cuff repair, often using healthy subscapularis or supraspinatus tendons as controls. For diseased rotator cuff muscle biopsies, ipsilateral healthy deltoid muscle served as a control. However, the presented findings were primarily derived from studies using matched controls (e.g. diseased vs. healthy supraspinatus tendon). Immunohistochemistry was applied across all studies, but marked variability existed in detection systems, antigen retrieval methods, and use of fluorescent co-labelling. Sample sizes were consistently small (typically < 20 per group), and criteria for defining expression fold-changes varied between studies.

### Overall molecular signatures

**Fig. 2:**
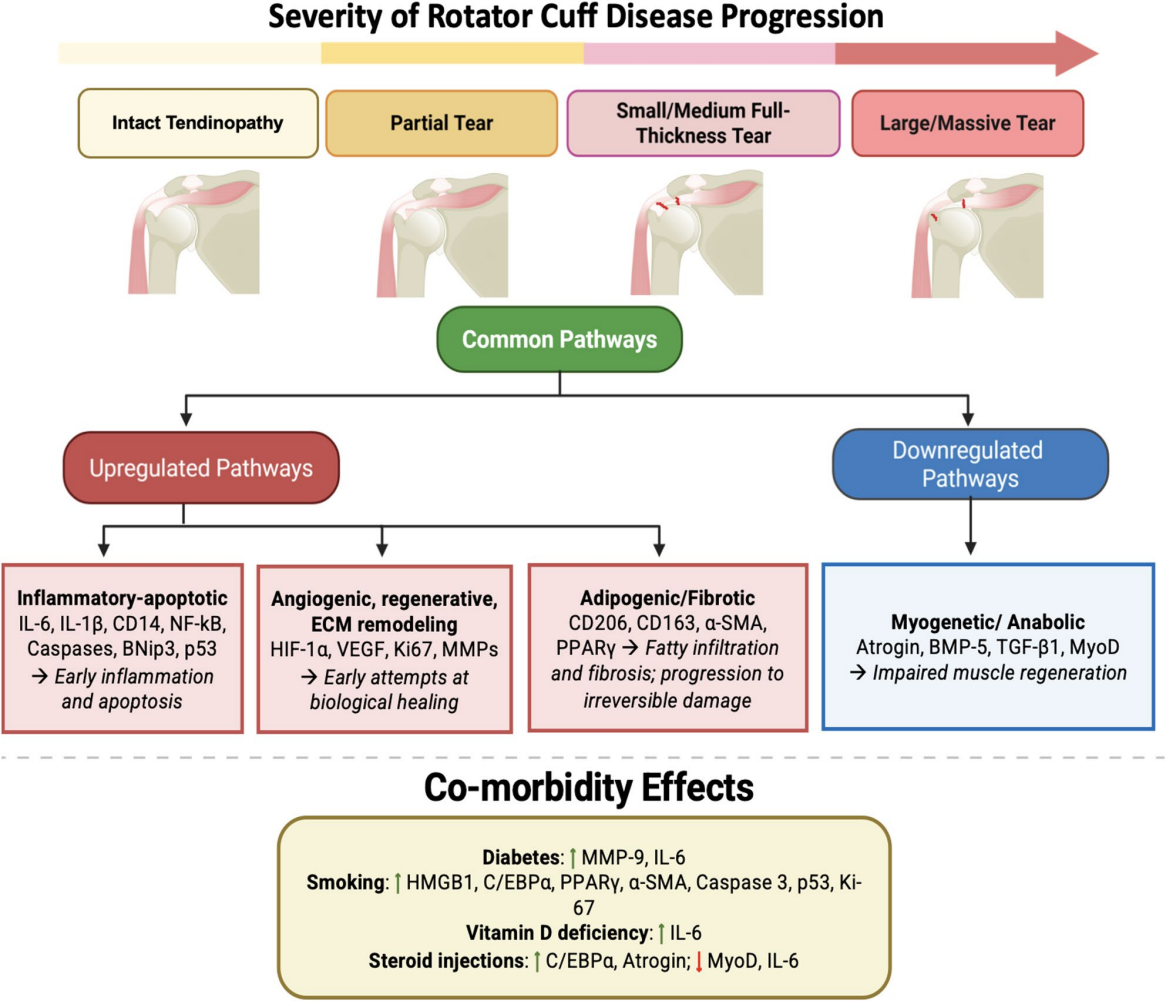
Stage-associated molecular signatures in rotator cuff disease and modifying effects of comorbidities

Despite the differences between included studies, consistent molecular signatures emerged. Broadly, diseased cuff tendon and muscle tissue showed upregulation of inflammatory markers (e.g. IL-6, IL-1β, CD14, NF-κB), matrix remodelling enzymes, extracellular matrix (ECM) proteins (fibronectin, vimentin, type I collagen), and hypoxic factors (HIF-1α). In parallel, studies reported upregulation of adipogenic proteins (PPARγ, C/EBPα), along with apoptotic (p21, p53, BNip3, caspases), atrophic (atrogin-1), and fibrotic markers (α-SMA, CD206, CD163). There was a general decrease in protective anti-inflammatory cytokines (TGF-β, IL-33). Protein expression was influenced by co-morbidities (diabetes, smoking, vitamin D deficiency, corticosteroid injections) (Fig. 2).

**Table 1:** Summary table of all included studies

Study characteristics		Patient demographics (*n*), sample characteristics, mean age					Direction of protein expression change in diseased rotator cuff samples (Immunohistochemistry)		
Study ID (Author & year of publication)	Location(s)	Patient samples (M/F)	Tear size/ characteristics	Mean age cases (years)	Control samples (M/F)	Mean age controls (years)	Up	Down	No difference
Benson et al. 2010 [21]	UK	24 (16 M/8F)	Partial thickness, small–massive full-thickness supraspinatus tears	49.3	3 (3 M/0F) Healthy subscapularis tendon	19.7	HIF-1α, BNip3 (in tears ≤Large)	BNip3 (massive tears)	–
Castagna et al. 2013 [22]	Italy, UK	13 (8 M/5F)	Chronic full-thickness supraspinatus tear	55	Internal controls	–	S-100	–	–
Chaudhury et al. 2016 [11]	UK	16	Small and large full-thickness tears	–	8 healthy supraspinatus/ subscapularis tendon	–	MMP-3 (small/large tears), BMP-5 (small tears), SAA (large tears)	–	–
Chung et al. 2017 [23]	South Korea	12 (6 M/6F) diabetic patients	Medium full-thickness supraspinatus tears	62.3 ± 9.9	12 (6 M/6F) non-diabetic patients	62.3 ± 9.9	MMP-9, IL-6	–	MMP-13, TNMD
Cole et al. 2001 [24]	UK	28 (14 M/14F)	Acute and chronic degenerative tears	Chronic: 65.1 Acute: 62.8	–	–	Amyloid P protein (more in chronic)	–	β2-microglobulin, prealbumin, light chains, amyloid A protein
Dakin et al. 2015 [12]	UK	42	Intact tendinopathy, full-thickness supraspinatus tears	Early: 52 ± 8 Full-thickness: 61 ± 6.5	12 heathy supraspinatus and subscapularis tendon	Healthy supraspinatus: 23 ± 3.8, Healthy subscapularis: 66 ± 8	CD14, CD68, IRF5, IDO-1, CD206, CD163 (greater increase in pain-free cases). FPR2/ALX, ChemR23 (in intact tendinopathy)	FPR2/ALX, ChemR23 (in full-thickness tears)	–
Dakin et al. 2017 [25]	UK	26–15 diseased and 11 post treatment	Small–massive full-thickness supraspinatus tears	55 ± 18.3	9 healthy supraspinatus, subscapular, hamstring	Healthy supraspinatus: 23 ± 3.8, Healthy subscapularis: 66 ± 8, Healthy hamstring: 25.5 ± 11	PDPN, CD14, CD68, CD248, CD106 (increased and persistent in diseased and post-treatment tissues). TLR4. FPR2/ALX, ChemR23 (early-stage disease), CD206 (in resolution of pain)	–	–
Dean et al. 2014 [26]	UK	20–12 GCI (6 M/6 F); 8 RCR (5 M/3 F)	GCI: supraspinatus tendinopathy (intact/partial tear) RCR: full-thickness supraspinatus tears	GCI: 49 ± 5 RCR: 58 ± 6	–	Paired comparison: (Week 0 vs. Week 7) samples	Glutamate, p53 (Both groups); HIF-1α (RCR); PCNA (RCR); NMDAR1 (GCI); mGluR1 (Both groups); mGluR7 (RCR); NK-1 (RCR); p75 (RCR); TrkA (RCR); PGP9.5 (RCR)	–	–
Dean et al. 2015 [27]	UK	18 (13 M/5F)	Painful and painless supraspinatus tendinopathy	Painful: 51 ± 8.2 Pain-free: 52 ± 7.8	–	Internal comparison	CD45, CD206, PGP9.5, mGluR2, KA1, LDH (increased in Painful vs. Pain-free)	mGluR7, VEGF (Decreased in Painful vs. Pain-free)	Glutamate, NMDAR1, mGluR1, mGluR3, mGluR4, mGluR5, mGluR8, PCNA
Franklin et al. 2014 [28]	UK	64 (39 M/25F)	Small–massive full-thickness supraspinatus tears	61	16 (14 M/2F)	23	Glutamate, NMDAR1, mGluR2, HIF1α (increased in L/M vs. S/M tears), α-2a adrenergic receptor (Increased in L/M vs. S/M tears)	mGluR7, NK-1, BDKRB2, TH, PGP9.5, Nav1.7, TRPA1, PCNA (decreased in tears vs. controls)	Substance P, VEGF, GAP43, p53
Frich et al. 2021 [29]	Denmark	22	Full-thickness supraspinatus tears	–	Ipsilateral deltoid	–	CD68 Adipophilin/Perilipin-2	–	–
Funakoshi et al. 2010 [30]	USA	7	Full-thickness supraspinatus tears	–	6 cadavers (4 M/2F)	–	Lubricin	–	–
Gigliotti et al. 2017 [31]	Canada	27 (20 M/7F)	Partial, full-thickness supraspinatus tears	56.6 ± 1.5	Ipsilateral deltoid	–	–	MyHC1	–
Goodier et al. 2016 [32]	UK	53	Tendinopathy, small–massive full-thickness tears	Tendinopathy: 54 Tears: 69.5	17 healthy supraspinatus and hamstring	Supraspinatus: 24 Hamstring: 31	pan-TGFβ, CD206, CD163 (massive tears)	TGFβ-1, TGFβ R1, TGFβ R2	–
Goodmurphy et al. 2003 [16]	USA, Canada	8	Small–large full-thickness tears	60.1	6 cadaveric supraspinatus tendons	70.2	von Willebrand factor (in tissue away from tear vs. near tear/control); Procollagen type I	–	–
Gotoh et al. 1997 [33]	Japan	16	Partial, full-thickness supraspinatus tears	59	6 cadaveric supraspinatus tendons	65	Interleukin-1β, Cathepsin D, MMP-1, CD68, UCHL-1	–	L-26
Gumina et al. 2013 [34]	Italy	63 (35 M/28F)	Small–massive full-thickness tears	65	29 normal suprascapular tendons	Internal comparison	NF-kB, p65	–	–
Gumina et al. 2021 [35]	Italy	43 (23 M/20F)	Small, large, massive full-thickness supraspinatus tears	63.5 ± 6.1	15 (8 M/7F) normal supraspinatus tendons	67.9 ± 7.1	Lamin A (highest in small tears)	Lamin A (decrease from small to massive tears)	–
Hawthorne et al. 2024 [36]	USA	42	Full-thickness supraspinatus tears	62.1 ± 13.65	–	–	–	–	p16INK4a (patients ≥ 60 yrs), p21 (patients < 60 yrs)
Hejbøl et al. 2024 [37]	Denmark	33 (24 M/9F)	Full-thickness supraspinatus tears	59.9	Ipsilateral deltoid	Internal comparison	MyoD, myogenin	–	–
Ibarra et al. 2025 [38]	USA	70 (38 M/32F)	Partial, small–massive full-thickness tears	55.1 ± 13.65	29 intact cuffs (16 M/13F)	45.9 ± 15.5	LipidTOX (full-thickness, especially massive)	–	Fibronectin, LipidTOX (partial vs. control)
Ki et al. 2021 [39]	South Korea	12 (3 M/9F)	Medium full-thickness supraspinatus tears with high FI	High FI: 65.8 ± 10.1	12 (7 M/5F) medium tears with low FI	60.0 ± 7.8	IL-1β, Myostatin (especially in High FI)	–	–
Kim et al. 2023 [40]	South Korea	12 (6 M/6F) with vitamin D deficiency	Medium full-thickness tears	61.9 ± 9.3	12 medium tears with sufficient vitamin D	62.0 ± 5.2	IL-6	–	Atrogin, IL-1β, MMP-9, p53, TNF-α, COL1, COL3
Lakemeier et al. 2010 [41]	Germany	33	Large full-thickness supraspinatus tears Patte grade 1–3	Patte 1: 61 Patte 2: 65 Patte 3: 69	6 normal supraspinatus tendons	–	HIF, VEGF, (significantly higher in Patte Stage 3 vs. Stages 1, 2, and control)	–	–
Lakemeier et al. 2011 [42]	Germany	33 (12 M/21F)	Large full-thickness tears Patte grade 1–3	Patte 1: 61 Patte 2: 65 Patte 3: 69	6 (3 M/3F) normal cuff tendons	56	MMP-1, MMP-9	MMP-3	–
Lee et al. 2013 [43]	South Korea	14	Torn supraspinatus tendon	57 ± 10.2	7 intact supraspinatus tendons	55 ± 9.45	Caspase 3/7, Caspase 8, Caspase 9, Cytochrome c	–	–
Lee et al. 2019 [44]	South Korea	12 (12 M/0F) smokers	Medium full-thickness supraspinatus tears	61.8 ± 5.1	12 (12 M/0F) non-smokers with medium tears	61.8 ± 6.9	HMGB1, C/EBPα, PPARγ, α-SMA	–	–
Lee et al. 2024 [45]	South Korea	12 (corticosteroid injections)	Medium full-thickness supraspinatus tears	63.0 ± 7.2	12 medium supraspinatus tears (no steroid)	62.8 ± 7.8	C/EBPα, Atrogin	MyoD, IL-6	–
Lohberger et al. 2016 [46]	Austria	9	Degenerative rotator cuff tears	68	Normal cuff tendon	Internal comparison	Vimentin	–	–
Longo et al. 2021 [47]	Italy	15 postmenopausal women	Partial, small–mass full-thickness supraspinatus tears	63.9 ± 7.1	9 men with partial, small–mass full-thickness supraspinatus tears	61.7 ± 9.0	ER-β, PR, ER-α (higher in women vs. men)	– (PR decreased with age in women)	–
Lundgreen et al. 2011 [48]	Norway, Canada	15 (10 M/5F)	Medium full-thickness supraspinatus tears	57.7	10 (5 M/5F) intact subscapularis tendon	43.9	Ki67, p53	–	–
Lundgreen et al. 2013 [49]	Norway, Canada	15	Full-thickness supraspinatus tears	58	9 partial thickness supraspinatus tears	54	–	CD56, Ki67, MHC1	Caspase 3
Lundgreen et al. 2014 [50]	UK	10 smokers	Full-thickness supraspinatus tears	52 ± 6.4	15 non-smokers with full-thickness supraspinatus tears	58 ± 5.8	Caspase 3, p53, Ki-67	–	–
Matthews et al. 2006 [51]	Australia	40 (23 M/17F)	Small–massive full-thickness supraspinatus tears	61.8	4 (4 M/0F)	20	PCNA, CD34, CD68, tryptase, CD45 (highest in small tears, decreasing with size) Amyloid, Chondroid metaplasia (more common in large/massive tears)	–	–
Millar et al. 2008 [52]	Australia	17	Torn supraspinatus tendons	57	10 intact subscapularis tendons	35	Caspase 3, Caspase 8	–	–
Millar et al. 2009 [53]	Australia	17	Torn supraspinatus tendons	57	10 intact subscapularis tendons	35	IL-18, IL-15, IL-6, MIF, Caspase 3, Caspase 8	–	–
Millar et al. 2010 [54]	UK, Australia	20	Small–massive full-thickness supraspinatus tears	57	10 intact subscapularis tendons	35	CD68, CD206, tryptase, CD3, CD34	–	–
Millar et al. 2012 [55]	UK, Australia	15	Torn supraspinatus tendons	55	10 intact subscapularis tendons	32	HIF-1α, Clusterin, Bcl-2	–	–
Mosca et al. 2017 [56]	UK	18	Painful early supraspinatus tendinopathy	45	6 healthy supraspinatus tendons	57	HIF-1α, S100A9 (diseased), HMGB1 (pain free)	IL-33	HMGB1 (diseased)
Oliva et al. 2009 [57]	Italy	5 (2 M/3F)	Full-thickness supraspinatus tears	60 ± 1	5 normal supraspinatus tendons (3 M/2F)	65 ± 1	Factor XIII	TG2	TG1, TG3
Nakama et al. 2006 [58]	Japan	30	Partial, full-thickness supraspinatus tears	56	Co-existing inflammatory subacromial synovium	Internal comparison	IL-6, IL-6 receptor, phosphorylated STAT3	–	CD20, CD45, CD68
Nakase et al. 2002 [59]	Japan	7 (5 M/2F)	Full-thickness rotator cuff tears	–	–	–	CDMP-1	–	–
Premdas et al. 2001 [60]	Sweden	8	Torn rotator cuff	49	–	–	α-SMA	–	–
Tillander et al. 2002 [61]	Australia	7 (5 M/2F)	Partial, full-thickness supraspinatus tears	57	9 (5 M/4F) intact cuff	28	Fibronectin	–	MMP-1
Wang et al. 2007 [62]	Australia	4 (2 M/2F)	Torn supraspinatus	61	4 normal subscapularis tendons	Internal comparison	Phosphorylated JNK, MMP1	–	–
Wu et al. 2011 [63]	Australia	30 (26 M/4F)	Chronic rotator cuff tears	57 ± 2	–	Internal comparison	Ubiquitin, α-SMA	–	–
Yuan et al. 2002 [64]	Australia	25	Torn supraspinatus tendons	61	6 normal subscapularis tendons	24	CD68 (sparse), fibroblast protein	–	–

M, Male; F, Female; RCT,  Rotator cuff tear; GCI,  Glucocorticoid injection; RCR, Rotator cuff repair; FI,  Fatty infiltration

## Intact tendinopathy

From eight intact tendinopathy studies, diseased tendons exhibited hypoxic, inflammatory, and apoptotic changes preceding macroscopic tearing. HIF-1α and its downstream pro-apoptotic effector BNip3 were upregulated, as well as proinflammatory cytokine IL-6 [21, 53, 55, 56]. IL-33, an anti-inflammatory cytokine, was reduced, promoting an inflammatory response [56]. Intact tendons demonstrated elevated expression of heat shock proteins, apoptotic mediators (Caspase-3, Caspase-8, BAX, Bcl2), and MMPs alongside reduced TGF-β1, TGFβR1, and TGFβR2 expression, implying impaired anabolic signalling [12, 25, 32, 52, 55]. Increased macrophage and stromal activation markers (CD14, CD68, PDPN, CD248) further reflected a persistent inflammatory, fibroproliferative milieu [25]. Painful intact tendinopathic samples showed upregulated CD45, CD206, PGP9.5, and mGluR2 compared with pain-free tendons, associated with enhanced inflammatory cell infiltration, macrophage activation, neuronal ingrowth, and pain sensitization [27].

## Partial-thickness tears

From five partial-thickness tear studies (*n* = 55), early structural disruption was characterised by hypoxic (BNip3 and HIF-1α) and inflammatory (IL-1β, Cathepsin D, CD68) signatures, with limited degeneration [21, 33]. The IL-6/STAT3 axis was activated within vascular and perivascular compartments, supporting a local pro-angiogenic and inflammatory response [58]. Despite these catabolic changes, muscle homeostasis remained relatively preserved: partial tears showed normal myofiber cross-sectional area, with no difference in fibrosis or lipogenesis to controls [38]. Increased CD56, Ki67 and MHC expression compared with full-thickness tears reflected maintained regenerative capacity [49]. Nevertheless, this severity stratum relied on the smallest number of studies.

### Small full-thickness tears

From eight small-tear studies (*n* = 108), tears demonstrated a combination of intense cellular activity and limited degeneration. HIF-1α and BNip3 were maximally expressed in small tears relative to intact tendinopathy, consistent with increased cellular stress in this disease stage [21]. Upregulation of MMP-3, and NF-κB p65 indicated activation of catabolic inflammatory pathways, while neo-angiogenesis, fibroblast proliferation (PCNA), and elevated Lamin A/C expression reflected robust tissue remodelling [11, 34, 35]. Small tears showed the highest CD68, tryptase (mast cell), CD45, and CD34 expression, consistent with an acute inflammatory–reparative phenotype that declined with tear enlargement [51]. Despite this, myofiber architecture and extracellular matrix organisation remained largely preserved [34, 38]. Neurochemical analyses revealed further glutamatergic dysregulation (mGluR2, NMDAR1, mGluR4), suggesting heightened nociceptive signalling [28].

### Medium full-thickness tears

From thirteen medium tear studies (*n* = 242), hypoxia and apoptotic stress was prominent, accompanied by elevated apoptotic indices (p53), signifying cellular injury and turnover [21, 41, 48]. VEGF expression was increased, supporting active angiogenesis [41]. Despite this, vascularity and inflammatory cell density (CD34, CD68, tryptase) began to decline relative to small tears, consistent with a shift from acute inflammation toward chronic remodelling [51]. Catabolic–inflammatory mediators (MMP-1, MMP-9, IL-6) were elevated, suggesting preferential matrix degradation and chronic inflammatory signalling.

In metabolic subgroups, diabetic and vitamin-D-deficient medium tears demonstrated exaggerated IL-6 and MMP-9 expression [23, 40]. Corticosteroid-exposed samples showed reduced IL-6 and MyoD, but increased C/EBPα and Atrogin, implicating myogenic suppression and adipogenic drift [45]. Smokers with medium tears showed heightened inflammation, fat accumulation, and fibrosis, underpinned by increased HMGB1, PPARγ, and α-SMA expression [44]. Myostatin and IL-1β were increased in fatty-infiltrated medium tears [39], reinforcing a catabolic–adipogenic milieu. Histologically, cellular proliferation (Ki67, PCNA) and vascularity were intermediate between small and large tears, while chondroid metaplasia became more prevalent, marking fibrocartilaginous transformation [48, 51]. Neuro-glutamatergic remodelling persisted [28].

### Large and massive full-thickness tears

In large (10 studies, *n* = 160) and massive (7 studies, *n* = 78) tear studies, hypoxia and apoptosis remained pronounced, but declined from their peak in smaller tears, suggesting loss of adaptive stress responses [21]. Nuclear stability markers (Lamin A) showed progressive depletion with increasing tear size [35]. Inflammatory cell populations (CD68, CD45, mast cells) and fibroblast proliferation (PCNA) declined with tear enlargement, marking a shift from active inflammation to a chronically fibrotic, low-cellularity state [51]. NF-κB p65 activation persisted at tear margins [34], while elevated M2 macrophage associated markers (CD206, CD163) co-expressing pan-TGFβ suggested profibrotic signalling [32]. Structurally, large-massive tears exhibited matrix derangement, with reduced vascularity, widespread chondroid metaplasia, and amyloid deposition [11, 51]. Procollagen I and MMP-9 were elevated [16, 42], indicating attempts at repair amid progressive tissue breakdown. Conversely, key anabolic mediators (TGFβ-1, TGFβR1, TGFβR2) were significantly suppressed, denoting impaired regenerative signalling [32]. Large-massive tears showed marked muscular atrophy and severe myosteatosis (LipidTOX accumulation), with adipogenic and fibrogenic replacement [38]. Dysregulated glutamatergic signalling persisted, while PGP9.5 fell sharply, suggesting axonal loss despite ongoing nociceptive activity [28] (Table 2).

**Table 2:** Summary of stage-associated protein expression patterns in human rotator cuff disease

Disease stage	Key proteins
Intact tendinopathy	Upregulated: HIF-1α, BNip3, IL-6, Caspase-3, Caspase-8, BAX, Bcl2, CD14, CD68, PGP9.5, mGluR2 (painful tendinopathy)
	Downregulated: IL-33, TGF-β
Partial tears	Upregulated: HIF-1α, BNip3, IL-6, IL-1β, Cathepsin D, CD68, CD56, Ki67, MHC
Small tears	Upregulated: HIF-1α, BNip3 *(maximal expression)*, MMP-3, NF-κB p65, PCNA, Lamin A/C, CD68, tryptase, CD45, CD34 *(maximal expression)*
Medium tears	Upregulated: VEGF, MMP-1, MMP-9, IL-6
	Downregulated: CD34, CD68, tryptase *(relative to small tears)*
Large and massive tears	Upregulated: CD206, CD163, pan-TGFβ
	Downregulated: PGP9.5, Lamin A/C, TGF-β1, TGFβR1, TGFβR2

“Maximal expression” indicates the disease stage at which a given protein showed the highest relative expression within an included study

### Risk of bias

All included studies were critically appraised using the Joanna Briggs Institute (JBI) Critical Appraisal Checklists appropriate to study design (Supplementary Tables 1 and 2). It is important to note that these assessments evaluate internal validity within individual studies, rather than the overall certainty, generalisability, or strength of the body of evidence. Forty-five studies were classified as analytical cross-sectional and two as quasi-experimental. Among the cross-sectional studies, forty-one (91%) were rated as low risk of bias and four (9%) as moderate risk. Studies classified as low risk demonstrated clearly defined inclusion criteria, standardised immunohistochemical outcome measures, appropriate consideration of confounders, blinded assessment, and appropriate statistical analyses. Studies rated as moderate risk were primarily limited by small sample sizes, absent control groups, or incomplete reporting of blinding procedures or confounder management. Both quasi-experimental studies were judged to have a moderate risk of bias, largely due to the absence of pre-intervention baseline measurements and limited external validity inherent to experimental models. Accordingly, “low risk of bias” ratings should not be interpreted as indicating high evidentiary strength or causal certainty, but rather as reflecting methodological rigour within the constraints of predominantly cross-sectional and heterogeneous study designs. These risk of bias assessments are interpreted alongside broader limitations of the literature, including reliance on observational methodologies, variability in immunohistochemical protocols, and substantial inter-study heterogeneity.

## Discussion

This systematic review synthesises immunohistochemical evidence from 47 human studies to conceptually infer the stage-specific proteomic landscape of rotator cuff degeneration. Despite methodological heterogeneity, convergent molecular patterns emerged at various disease stages.

### Proteomic trajectory of degeneration

This synthesis explores protein expression at various rotator cuff disease stages. Intact tendinopathy and partial tears demonstrated a metabolically active phenotype characterized by upregulation of hypoxia-inducible factors (HIF-1α, BNip3), pro-inflammatory cytokines (IL-6, IL-1β, NF-κB), and matrix-degrading enzymes. Critically, these tissues retained markers of regenerative capacity, including cellular proliferation (Ki67, CD56) and vascularity, suggesting residual healing potential. Medium-sized tears exhibited both active remodelling and emerging adipogenic drift (PPARγ, C/EBPα). In contrast, large and massive tears exhibited depletion of anabolic growth factors (TGF-β1, BMP-5, TGFβR1/R2), loss of nuclear mechano-transduction capacity (Lamin A/C), and increased fibrotic markers. This was accompanied by hallmarks of structural degeneration, including amyloid deposition, chondroid metaplasia, and myosteatosis [24, 51]. However, the exact molecular thresholds defining the transition from intact tendinopathy to massive tears remain undefined and likely vary between patients based on age, chronicity, and comorbidities. The cross-sectional nature of immunohistochemical studies precludes definitive conclusions about causality and temporal ordering. The described proteomic trajectory should therefore be interpreted as an inferential model based on associations with disease severity, rather than a validated biological progression.

### Inflammatory dynamics and macrophage polarization

An important observation is the differences in macrophage-associated marker expression at different disease stages. Partial and small tears demonstrated increased expression of markers associated with pro-inflammatory macrophage activity (IL-1β, IL-6, and CD68). Large-massive tears exhibited higher expression of markers linked to fibrotic and reparative macrophage-associated programs (CD206, CD163, α-SMA, and TGF-β). At a conceptual level, this pattern is consistent with wound-healing frameworks in which coordinated transitions from inflammatory to reparative immune signalling are required for tissue repair [65]. However, this interpretation should be regarded as putative rather than definitive. Notably, most studies relied on single marker identification of macrophages, which limits the assignment of discrete M1 or M2 phenotypes and oversimplifies the underlying cellular heterogeneity. Contemporary immunology increasingly recognises macrophage activation as a plastic, context-dependent continuum rather than a binary M1/M2 state; macrophages frequently co-express markers traditionally assigned to both phenotypes [66]. The M1/M2 paradigm is therefore best interpreted here as a heuristic describing broad inflammatory tendencies, rather than evidence of fixed macrophage polarisation states. Supporting this cautious interpretation, in vitro and translational studies suggest that immune-regulatory pathways, including CD200 signalling and carbonic anhydrase activity, influence inflammatory balance and matrix remodelling in rotator cuff pathology [67, 68].

Comorbidities substantially modified the inflammatory landscape. Diabetes and vitamin D deficiency intensified IL-6 [23, 40], while corticosteroids suppressed myogenic signalling (MyoD) and promoted adipogenic differentiation (C/EBPα) [45]. Smoking amplified oxidative stress (HMGB1) and fibrogenesis (α-SMA) [44]. These findings may support preoperative strategies, including glycaemic control, smoking cessation, and vitamin D repletion [40, 69, 70]. However, these therapies require further prospective validation.

### Matrix homeostasis, mechano-transduction, and hypoxia

Extracellular matrix (ECM) remodelling was apparent at various disease stages. Small and medium tears exhibited increased matrix turnover with elevated MMP-3, BMP-5, and fibroblast proliferation, alongside neoangiogenic responses (VEGF). Large tears demonstrated persistent MMP-9 and procollagen I expression despite depleted vascularity, suggesting futile repair attempts. Progressive downregulation of Lamin A/C, paralleled tear enlargement [35], potentially reflecting impaired adaptive responses to mechanical loading. Concurrently, persistent HIF-1α activation transitioned from adaptive to maladaptive, consistent with chronic hypoxia as a central pathogenic driver [21].

### Neural sensitization and metabolic dysfunction

Two studies identified dysregulated glutamatergic signalling in rotator cuff tears [27, 28]. These alterations may underpin persistent pain despite anatomical repair, framing rotator cuff disease as both a structural and neurochemical disorder. Similarly, increased PGP9.5 and substance P in intact tendinopathy contrasted with axonal depletion in massive tears, suggesting initial neurogenic inflammation followed by denervation [28]. Adipogenic factors were upregulated in large-massive tears, linking metabolic dysregulation to fatty infiltration. This adipogenic drift likely reflects fate-switching of fibro-adipogenic progenitors rather than trans differentiation of mature tenocytes [71]. However, lineage-tracing studies in humans are lacking.

### Translational implications and clinical applications

This synthesis suggests candidate protein panels that could inform rotator cuff tear classifications (e.g. reparable vs. irreparable), which are currently limited by lack of consensus [72]. Clinical outcome studies demonstrate that early arthroscopic repair is associated with superior functional outcomes and lower re-tear rates in traumatic tears, whereas comparable timing benefits are not consistently observed in non-traumatic disease [73]. In this context, proteomic classification may offer a biological framework to support more patient-specific decisions regarding the timing and suitability of surgical intervention.

Our findings may support a molecular staging system that integrates protein signatures with tear dimensions. Candidate biomarkers including IL-6, MMP-9, BNip3, HIF-1α, PPARγ, α-SMA, and TGF-β1 emerged at various disease stages, though their predictive validity for postoperative outcomes has not been prospectively tested. Accordingly, immunohistochemical biomarkers should not be considered decision-making tools at present, but rather candidates for future validation in longitudinal studies. Additionally, given that structural abnormalities on imaging in rotator cuff disease may not be associated with clinical outcomes [74, 75], future investigations should prioritise linking such immunohistochemical markers to patient-relevant outcomes, such as pain or function, rather than anatomical features alone.

The concept of “molecularly profiling” surgical biopsies is appealing but faces practical barriers. Immunohistochemical profiling requires laboratory processing that may delay surgical decision-making, and its incremental prognostic value over established clinical predictors remains unproven. At present, immunohistochemical profiling is impractical for routine clinical decision-making. It should therefore be viewed primarily as a hypothesis-generating research tool, not an adjunct or replacement for current clinical algorithms.

Regarding therapeutic implications, this synthesis supports biologically plausible strategies that warrant further investigation. These proposed therapeutic strategies are hypothesis driven avenues rather than emerging standards of care.

Phase-targeted immunomodulation could restore the natural sequence of tendon healing, involving a transient activation of M1 macrophages to clear necrotic tissue followed by M2 polarization to promote healing [76]. Anti-fibrotic therapies represent another promising avenue, focused on inhibiting myofibroblasts or attenuating aberrant TGF-β signalling to limit excessive scar formation [77]. Metabolic interventions, such as PPARγ modulators or GLP-1 receptor agonists, could theoretically counteract adipogenic drift within the musculotendinous unit [78, 79]. Finally, matrix augmentation approaches, including platelet-rich plasma, acellular scaffolds, or targeted growth factor delivery, could re-establish anabolic signalling and improve the biological microenvironment for repair [80]. Nonetheless, the use of protein biomarkers to guide rotator cuff interventions is hypothesis-generating, as the clinical utility remains unproven. Future mechanistic validation is required.

### Limitations

This review has several limitations that constrain interpretation. A major limitation is the heterogeneity in rotator cuff disease classification systems (e.g. Cofield, Patte) and control tissues across included studies. Disease severity was variably defined using tear size, tendon retraction, fatty infiltration, or symptom duration, while control tissues ranged from intact supraspinatus to subscapularis tendons, deltoid muscle, or hamstring specimens. Pertaining to these controls, intrinsic differences in vascularity, loading environment, and developmental origin of control tissues may confound protein expression comparisons. However, the conclusions presented were primarily derived from studies using matched controls (e.g. diseased vs. healthy supraspinatus tendon).

Such heterogeneity may confound molecular comparisons attributed to disease stage, as observed protein expression patterns may reflect variation in anatomical site, tissue quality, or control selection rather than true biological progression. Thus, the structured proteomic “trajectory” discussed (from intact tendinopathy to massive tears) should be interpreted as an inferential framework rather than definitive evidence of linear molecular progression. An additional limitation relates to the inherent constraints of immunohistochemistry. Immunohistochemical analysis is semi-quantitative, cross-sectional, and highly sensitive to methodological variation, including antibody selection, antigen retrieval protocols, tissue fixation, and imaging thresholds. As a result, reported differences in protein expression across studies should be interpreted as directional trends within individual studies, rather than as standardized quantitative differences that are directly comparable across the literature. Accordingly, this review focuses on the consistency of within-study expression patterns across disease stages, rather than attempting quantitative cross-study comparison of staining intensity or signal magnitude. Finally, most investigations focused on a single protein or small group of markers. Because immunohistochemistry examines limited targets, it cannot capture the full proteomic landscape or account for protein–protein interactions or subcellular localization dynamics.

### Future research directions

To progress from descriptive observations to clinically actionable molecular diagnostics, several key research priorities must be addressed. First, standardized molecular phenotyping is essential. A consensus immunohistochemical panel with validated antibodies, internal positive controls, and uniform quantification protocols could enable reliable cross-study comparison. Second, integration of high-throughput proteomics could complement conventional immunohistochemistry: mass spectrometry can profile thousands of proteins simultaneously, revealing novel biomarkers. Multi-omics integration that combines transcriptomic, proteomic, and metabolomic data may clarify regulatory networks underlying tendon degeneration. Third, prospective validation cohorts are needed to confirm the prognostic relevance of candidate proteins. Large, multicentre studies should correlate preoperative rotator cuff protein profiles with clinical outcomes such as re-tear rate, pain, and functional recovery. Finally, cost-effectiveness analyses are necessary to determine whether molecular profiling improves patient outcomes sufficiently to justify the additional expense, or whether current clinical algorithms can achieve comparable prognostic accuracy.

## Conclusions

This systematic review explores protein expression across the various stages of human rotator cuff disease, synthesizing immunohistochemical evidence from 47 studies. Despite substantial heterogeneity, molecular patterns emerged: partial and small tears exhibited inflammatory-hypoxic-apoptotic signatures with retained regenerative capacity, while large-massive tears demonstrated anabolic suppression, fibrosis, and adipogenic transformation. Medium tears exhibited mixed reparative and degenerative features. These findings may support a molecular stage-specific interpretation of rotator cuff disease, beyond just discrete anatomical pathology. However, while this molecular framework offers mechanistic insight into rotator cuff degeneration, its clinical application remains speculative. Prospective studies correlating tissue-level protein expression with clinical outcomes are required before molecular profiling can be considered to inform patient-specific decision making.

## Supplementary Information

Below is the link to the electronic supplementary material.

Supplementary Material 1

Supplementary Material 2

Supplementary Material 3

## Data Availability

No datasets were generated or analysed during the current study.

## Abbreviations

RC: Rotator cuff
ECM: Extracellular matrix
MRI: Magnetic resonance imaging
PRISMA: Preferred reporting items for systematic reviews and meta–analyses
JBI: Joanna Briggs Institute
HIF: 1α–Hypoxia–inducible factor–1 alpha
BNip3: BCL2 interacting protein 3
IL: 6–Interleukin–6
IL: 1β–Interleukin–1 beta
IL: 33–Interleukin–33
MMP: 1/3/9–Matrix metalloproteinases–1,–3,–9
CD34, CD14, CD45, CD56, CD68, CD163, CD206, CD248: Cluster of differentiation molecules
NF: κB–Nuclear factor kappa–light–chain–enhancer of activated B cells
PPARγ: Peroxisome proliferator–activated receptor gamma
C/EBPα: CCAAT/enhancer–binding protein alpha
TGF: β1–Transforming growth factor–beta 1
TGFβR1/R2: Transforming growth factor–beta receptor 1/2
TG1: Transglutaminase–1
TG2: Transglutaminase–2
TG3: Transglutaminase–3
BMP: 5–Bone morphogenetic protein–5
α: SMA–Alpha–smooth muscle actin
PCNA: Proliferating cell nuclear antigen
VEGF: Vascular endothelial growth factor
HMGB1: High mobility group box 1
FABP4: Fatty acid binding protein–4
MyoD: Myogenic differentiation 1
PGP9.5: Protein gene product 9.5
BAX: BCL2–associated X protein
Bcl2: B–cell lymphoma 2
HSP: Heat shock proteins
Caspase: 3, Caspase–8–Cysteine–aspartic proteases
p53: Tumour protein p53
p21: Cyclin–dependent kinase inhibitor 1
mGluR2/4: Metabotropic glutamate receptor 2/4
NMDAR1: N–methyl–D–aspartate receptor 1
HRP: DAB–Horseradish peroxidase–diaminobenzidine
ABC: Avidin–biotin complex
LSAB: Labelled streptavidin–biotin
IHC: Immunohistochemistry
FAPs: Fibro–adipogenic progenitors
GLP: 1RA–Glucagon–like peptide–1 receptor agonist
