# Supplementary material for: Proteomic trajectories in human rotator cuff degeneration: a systematic review of immunohistochemical studies
Source: J Orthop Surg Res. 2026 Feb 9;21:192. doi: 10.1186/s13018-026-06735-1 (PMC12983771; doi:10.1186/s13018-026-06735-1)
Supplement: Supplementary file 1 — Supplementary Material 1 [file 13018_2026_6735_MOESM1_ESM.docx]

| **Study** | **Question 1** | **Question**  **2** | **Question 3** | **Question**  **4** | **Question 5** | **Question 6** | **Question**  **7** | **Question**  **8** | **Question**  **9** | **Overall quality** |
| --- | --- | --- | --- | --- | --- | --- | --- | --- | --- | --- |
| Dean et al., 2014 | Yes | Yes | Yes | No | No | Yes | Yes | Yes | Yes | Moderate risk of bias |
| Lohberger et al., 2016 | Yes | Yes | Yes | Yes | No | Yes | Yes | Yes | Yes | Moderate risk of bias |

**Supplementary Table 2 – results of risk of bias assessment for quasi-experimental studies**
