# Supplementary material for: Proteomic trajectories in human rotator cuff degeneration: a systematic review of immunohistochemical studies
Source: J Orthop Surg Res. 2026 Feb 9;21:192. doi: 10.1186/s13018-026-06735-1 (PMC12983771; doi:10.1186/s13018-026-06735-1)
Supplement: Supplementary file 2 — Supplementary Material 2 [file 13018_2026_6735_MOESM2_ESM.docx]

| **Study** | **Question 1** | **Question**  **2** | **Question 3** | **Question**  **4** | **Question 5** | **Question 6** | **Question**  **7** | **Question**  **8** | **Overall quality** |
| --- | --- | --- | --- | --- | --- | --- | --- | --- | --- |
| Benson et al., 2010 | Yes | Yes | Yes | Yes | Yes | No | Yes | Yes | Moderate risk of bias |
| Castagna et al., 2013 | Yes | Yes | Yes | Yes | Yes | Yes | Yes | Yes | Low risk of bias |
| Chaudhury et al., 2016 | Yes | Yes | Yes | Yes | Yes | Yes | Yes | Yes | Low risk of bias |
| Chung et al., 2017 | Yes | Yes | Yes | Yes | Yes | Yes | Yes | Yes | Low risk of bias |
| Cole et al., 2001 | Yes | Yes | Yes | Yes | Yes | No | Yes | Yes | Moderate risk of bias |
| Dakin et al., 2015 | Yes | Yes | Yes | Yes | Yes | Yes | Yes | Yes | Low risk of bias |
| Dakin et al., 2017 | Yes | Yes | Yes | Yes | Yes | Yes | Yes | Yes | Low risk of bias |
| Dean et al., 2015 | Yes | Yes | Yes | Yes | Yes | Yes | Yes | Yes | Low risk of bias |
| Franklin et al., 2014 | Yes | Yes | Yes | Yes | Yes | Yes | Yes | Yes | Low risk of bias |
| Frich et al., 2021 | Yes | Yes | Yes | Yes | Yes | Yes | Yes | Yes | Low risk of bias |
| Funakoshi et al., 2010 | Yes | Yes | Yes | Yes | Yes | Yes | Yes | Yes | Low risk of bias |
| Gigliotti et al., 2017 | Yes | Yes | Yes | Yes | Yes | Yes | Yes | Yes | Low risk of bias |
| Goodier et al., 2016 | Yes | Yes | Yes | Yes | Yes | Yes | Yes | Yes | Low risk of bias |
| Goodmurphy et al., 2003 | Yes | Yes | Yes | Yes | Yes | Yes | Yes | Yes | Low risk of bias |
| Gotoh et al., 1997 | Yes | Yes | Yes | Yes | Yes | Yes | Yes | Yes | Low risk of bias |
| Gumina et al., 2013 | Yes | Yes | Yes | Yes | Yes | Yes | Yes | Yes | Low risk of bias |
| Gumina et al., 2021 | Yes | Yes | Yes | Yes | Yes | Yes | Yes | Yes | Low risk of bias |
| Hawthorne et al., 2024 | Yes | Yes | Yes | Yes | Yes | Yes | Yes | Yes | Low risk of bias |
| Hejbøl et al., 2024 | Yes | Yes | Yes | Yes | Yes | Yes | Yes | Yes | Low risk of bias |
| Ibarra et al., 2025 | Yes | Yes | Yes | Yes | Yes | Yes | Yes | Yes | Low risk of bias |
| Ki et al., 2021 | Yes | Yes | Yes | Yes | Yes | Yes | Yes | Yes | Low risk of bias |
| Kim et al., 2023 | Yes | Yes | Yes | Yes | Yes | Yes | Yes | Yes | Low risk of bias |
| Lakemeier et al., 2010 | Yes | Yes | Yes | Yes | Yes | Yes | Yes | Yes | Low risk of bias |
| Lakemeier et al., 2011 | Yes | Yes | Yes | Yes | Yes | Yes | Yes | Yes | Low risk of bias |
| Lee et al., 2013 | Yes | Yes | Yes | Yes | Yes | Yes | Yes | Yes | Low risk of bias |
| Lee et al., 2019 | Yes | Yes | Yes | Yes | Yes | Yes | Yes | Yes | Low risk of bias |
| Lee et al., 2024 | Yes | Yes | Yes | Yes | Yes | Yes | Yes | Yes | Low risk of bias |
| Longo et al., 2021 | Yes | Yes | Yes | Yes | Yes | Yes | Yes | Yes | Low risk of bias |
| Lundgreen et al., 2011 | Yes | Yes | Yes | Yes | Yes | Yes | Yes | Yes | Low risk of bias |
| Lundgreen et al., 2013 | Yes | Yes | Yes | Yes | Yes | Yes | Yes | Yes | Low risk of bias |
| Lundgreen et al., 2014 | Yes | Yes | Yes | Yes | Yes | Yes | Yes | Yes | Low risk of bias |
| Matthews et al., 2006 | Yes | Yes | Yes | Yes | Yes | Yes | Yes | Yes | Low risk of bias |
| Millar et al., 2008 | Yes | Yes | Yes | Yes | Yes | Yes | Yes | Yes | Low risk of bias |
| Millar et al., 2009 | Yes | Yes | Yes | Yes | Yes | Yes | Yes | Yes | Low risk of bias |
| Millar et al., 2010 | Yes | Yes | Yes | Yes | Yes | Yes | Yes | Yes | Low risk of bias |
| Millar et al., 2012 | Yes | Yes | Yes | Yes | Yes | Yes | Yes | Yes | Low risk of bias |
| Mosca et al., 2017 | Yes | Yes | Yes | Yes | Yes | Yes | Yes | Yes | Low risk of bias |
| Oliva et al., 2007 | Yes | Yes | Yes | Yes | Yes | No | Yes | Yes | Low risk of bias |
| Nakama et al., 2006 | Yes | Yes | Yes | Yes | Yes | Yes | Yes | Yes | Moderate risk of bias |
| Nakase et al., 2002 | Yes | Yes | Yes | Yes | Yes | Yes | Yes | Yes | Low risk of bias |
| Premdas et al., 2001 | Yes | Yes | Yes | Yes | Yes | Yes | Yes | Yes | Moderate risk of bias |
| Tillander et al., 2002 | Yes | Yes | Yes | Yes | Yes | Yes | Yes | Yes | Low risk of bias |
| Wang et al., 2007 | Yes | Yes | Yes | Yes | Yes | Yes | Yes | Yes | Low risk of bias |
| Wu et al., 2011 | Yes | Yes | Yes | Yes | Yes | Yes | Yes | Yes | Low risk of bias |
| Yuan et al., 2002 | Yes | Yes | Yes | Yes | Yes | Yes | Yes | Yes | Low risk of bias |

**Supplementary Table 1 – results of risk of bias assessment for cross-sectional studies**
