# Supplementary material for: Proteomic trajectories in human rotator cuff degeneration: a systematic review of immunohistochemical studies
Source: J Orthop Surg Res. 2026 Feb 9;21:192. doi: 10.1186/s13018-026-06735-1 (PMC12983771; doi:10.1186/s13018-026-06735-1)
Supplement: Supplementary file 3 — Supplementary Material 3 [file 13018_2026_6735_MOESM3_ESM.docx]

Medline (via Pubmed)

(("Rotator Cuff"[MeSH] OR "Rotator Cuff Injuries"[MeSH] OR "Supraspinatus"

OR "Infraspinatus"[tiab] OR "Subscapularis"[tiab] OR "Teres Minor"[tiab]

OR "rotator cuff"[tiab] OR supraspinatus[tiab] OR infraspinatus[tiab]

OR subscapularis[tiab] OR "teres minor"[tiab])

AND

(("Immunohistochemistry"[MeSH] OR "Immunohistochemical"[tiab]

OR "immunohistochemistry"[tiab] OR "immunohistochemical staining"[tiab]

OR "immunofluorescence"[tiab] OR "immunofluorescent"[tiab])

OR

("Proteins"[MeSH] OR "Protein Expression"[tiab] OR protein*[tiab]

OR cytokine*[tiab] OR biomarker*[tiab] OR transcription factor*[tiab])))

Embase (via Ovid)

1. ('rotator cuff'.ti,ab,kw.

OR supraspinatus.ti,ab,kw.

OR infraspinatus.ti,ab,kw.

OR subscapularis.ti,ab,kw.

OR 'teres minor'.ti,ab,kw.)

2. ('rotator cuff tear'.ti,ab,kw.

OR 'rotator cuff injury'.ti,ab,kw.

OR tendinopathy.ti,ab,kw.)

3. 1 OR 2

4. immunohistochemistry.ti,ab,kw.

OR immunohistochemical.ti,ab,kw.

5. immunofluorescence.ti,ab,kw.

OR immunofluorescent.ti,ab,kw.

6. 4 OR 5

7. 3 AND 6

Cochrane

("rotator cuff" OR supraspinatus OR infraspinatus OR subscapularis OR "teres minor")

AND

(immunohistochemistry OR immunohistochemical OR immunofluorescence)

AND

(protein OR proteins OR cytokine OR biomarker)
